# Distinct laccase expression and activity profiles of *Trametes versicolor* facilitate degradation of benzo[a]pyrene

**DOI:** 10.3389/fbioe.2023.1264135

**Published:** 2023-09-21

**Authors:** Yueming Sun, Ying Li, Hong Liang, Ming Li, Ye Liu, Litao Wang, Weijian Lai, Teng Tang, Yongzhao Diao, Yuhong Bai, Christian Isak Jørgensen, Wanghui Xu, Dawen Gao

**Affiliations:** ^1^ Novozymes (China) Investment Co., Ltd., Beijing, China; ^2^ Centre for Urban Environmental Remediation, Beijing University of Civil Engineering and Architecture, Beijing, China; ^3^ Beijing Energy Conservation and Sustainable Urban and Rural Development Provincial and Ministry Co-construction Collaboration Innovation Center, Beijing University of Civil Engineering and Architecture, Beijing, China; ^4^ Novozymes A/S, Lyngby, Denmark

**Keywords:** polycyclic aromatic hydrocarbon (PAH), benzo[a]pyrene (BaP), *Trametes versicolor*, laccase, multi-omics, soil remediation

## Abstract

A *Trametes versicolor* isolate from the Changbai Mountain showed promising activity in degrading benzo[a]pyrene (BaP), which is a high molecular weight (HMW) polycyclic aromatic hydrocarbon (PAH) compound. It was hypothesized that the *T. versicolor* isolate encode BaP-degrading enzymes, among which laccase is mostly sought after due to significant commercial potential. Genome of the *T. versicolor* isolate was sequenced and assembled, and seven laccase homologues were identified (*TvLac1-7*) as candidate genes potentially contributing to BaP degradation. In order to further identify the BaP responsive laccases, time-course transcriptomic and proteomic analyses were conducted in parallel on the *T. versicolor* isolate upon BaP treatment. Homologous laccases showed distinct expression patterns. Most strikingly, TvLac5 was rapidly induced in the secreted proteomes (secretomes), while TvLac2 was repressed. Recombinant laccase expression and biochemical characterization further showed corresponding enzymatic activity profiles, where TvLac5 was 21-fold more effective in BaP degradation compared to TvLac2. Moreover, TvLac5 also showed 3.6-fold higher BaP degrading activity compared to a commercial laccase product of *T. versicolor* origin. Therefore, TvLac5 was concluded to be a BaP-responsive enzyme from *T. versicolor* showing effective BaP degradation activity.

## 1 Introduction

Polycyclic aromatic hydrocarbons (PAHs) is a class of organic compounds containing two or more unsubstituted benzene rings. PAHs are hazardous organic contaminants highly resistant to degradation. Industrial PAHs are mainly generated due to incomplete combustion of organic materials (e.g., at petroleum refineries and wood processing sites). The ultimate sink of PAHs is the sediment or soil, where PAHs could remain for months or even years leading to destructive ecological consequences (Roslund et al., 2018). High molecular weight (HMW) PAHs are particularly toxic and can be carcinogenic to humans (Mastrangelo et al.,1996). Physical, chemical, and biological methods (Kuppusamy et al., 2017) have been developed to facilitate PAH degradation, including biocatalyst-assisted approaches utilizing enzyme products.

*Trametes versicolor* is a ligninolytic fungus that is capable of degrading lignin with its secreted enzyme system, including lignin peroxidases (LiP), manganese peroxidases (MnP) and laccases. It has been reported that the lignin-degrading enzymes could also function on a wide range of organic pollutants in soil including PAHs, thereby providing promising enzyme candidates for developing effective biocatalysts for PAH degradation (Zhuo and Fan, 2021). Among these enzymes, laccase shows the highest commercial potential, as the laccase-catalyzed reaction does not require addition of hydrogen peroxide.

A *T. versicolor* isolated from the Changbai Mountain showed promising activity against benzo[a]pyrene (BaP), a high molecular weight (HMW) 5-ring PAH compound (Wen et al., 2011). The reference genome of *T. versicolor* (RefSeq accession: GCF_000271,585.1) encodes seven copies of laccase genes, some of which were expressed and tested against selected substrates showing various activities (Koschorreck et al., 2008). Therefore, it is hypothesized that multi-copy laccases encoded in *T. versicolor* are not genetically redundant and show distinct expression and activity profiles responsive to environmental signals.

A multi-omics approach was adopted in this study to elucidate laccase expression profiles in the *T. versicolor* isolate induced by benzo[a]pyrene (BaP), where BaP was both the inducer and the substrate. Transcriptomic and proteomic sequencing were conducted in parallel at different timepoints after BaP treatment, for the purpose of identifying differentially expressed laccases at secreted protein level and the corresponding transcriptional regulation patterns. The upregulated laccase responsive to BaP treatment was hypothesized to show significant activity against BaP and was subsequently validated by heterologous expression and biochemical characterization.

## 2 Methods

### 2.1 Strain identification

The isolate was cultivated on potato dextrose agar (PDA; Difco™, Becton, Dickinson and Company, Sparks, MD, USA) at 28 °C. The morphological and microscopic characteristics were observed for identification. After 5-day cultivation, the mycelia were collected, and total genomic DNA was extracted. The ITS gene was amplified using the primer pairs ITS4 and ITS5 (White et al., 1990) followed by Sanger sequencing. The ITS sequence was BLAST against the NCBI nucleotide collection, based on which the strain was identified.

### 2.2 Genome assembly

Genome sequencing, assembly and annotation was conducted by Azenta Life Sciences. Briefly, the sequencing library was prepared from genomic DNA extracted from fresh mycelia, and pair-end (PE150) sequenced on the illumina Novaseq System. The reads were quality filtered and then assembled using velvet, gapfilled with SSPACE (Boetzer et al., 2011) and GapFiller (Nadalin et al.,2012). Protein-coding genes were identified using Augustus (Stanke and Morgenstern, 2005), and annotated using NCBI BLAST against the nr database. Gene functions and pathways were then annotated using GO (Gene Ontology) database and KEGG (Kyoto Encyclopedia of Genes and Genomes) database respectively.

### 2.3 Benzo[a]pyrene treatment and degradation analysis

The *T. versicolor* isolate was inoculated on potato dextrose agar, and the mycelia were subsequently inoculated in bran extract liquid media (Bran 20 g/L, NH_4_Cl 0.44 g/L, KH_2_PO_4_ 0.2 g/L, MgSO_4_ 0.05 g/L, CaCl_2_ 0.01 g/L, Tween 80 1.0 g/L, and 0.1% of inorganic solution (pH 7) containing anhydrous MgSO_4_ 1.47 g/L, MnSO_4_ 0.5 g/L, NaCl 1.0 g/L, FeSO_4_-7H_2_O 0.1 g/L, CaCl_2_ 0.082 g/L, ZnSO_4_ 0.1 g/L, CuSO_4_-5H_2_O 0.01 g/L, KAl(SO_4_)_2_ 0.01 g/L, H_3_BO_3_ 0.01 g/L, NaMoO_4_ 0.01 g/L) and grown for 5 days at 30°C and 160 rpm, before benzo[a]pyrene was added to the final concentration of 10 mg/L. Sterile water was added to the uninduced controls. The uninduced and induced cultures were sampled 1, 4 (logarithmic phase) and 11 days (stationary phase) after the BaP treatment in quadruplets.

The fermentation broth after BaP induction was centrifuged at 4000 rpm for 5 min, and the supernatant was extracted 1:1 (volume ratio) with dichloromethane three times. In parallel, the mycelium was harvested, homogenized, and extracted with 5 mL dichloromethane followed by 30-min ultrasonication and centrifugation (4000 rpm, 5 min) to remove debris. The fermentation broth extract and the mycelium extract were then combined with dichloromethane evaporated, redissolved in 25 mL acetonitrile, and filtered through a 0.22 μm organic membrane.

Residual BaP was separated by Agilent 1260 Infinity HPLC system with an Eclipse Plus C18 (4.6 mm × 150 mm, 5 μm) at 30°C. The isocratic elution solvents were composed of acetonitrile (85%) and water (15%). The elution rate was set at 1 mL/min, injection volume was 10 μL. And the detection wavelength of benzo[a]pyrene was 290 nm.

Degradation rate of BaP was calculated by the formula: $\frac{C_{0}-C_{i}}{C_{0}}\times 100$

C_0_ and C_i_ represent residual BaP concentration measured at timepoint 0 and the sampling timepoint, respectively.

### 2.4 Transcriptomic analysis

Transcriptome sequencing and analysis was conducted by Azenta Life Sciences. Briefly, the sequencing libraries were prepared from the sampled mycelium materials described above, and were pair-end (PE150) sequenced on the illumina Novaseq System. The genome assembled in this study was used as the reference. Quality filtered and trimmed reads were aligned to the reference genome using Hisat2 (v2.0.1). Gene expression levels were quantified using HTSeq (v0.6.1), and differential expression analysis was conducted using DESeq2. A fold-change of 2-fold and an adjusted *p*-value of 0.05 were adopted as the threshold for calling significant DEGs.

### 2.5 Proteomic analysis

Supernatant (500 µL) of each sample was precipitated by 3 volumes of acetone at −20°C for 2 h. Pellets were re-dissolved in 300 µL DOC solution (1% DOC, 10 mM TCEP, 40 mM CAA, 100 mM Tris at pH 8.5) at 95°C for 5 min, centrifuged at 14,000 *g* for 5 min, and supernatants were recovered and assayed for protein concentration. The sample buffer was then replaced with 400 µL 50 mM NH_4_HCO_3_ buffer using a 10 kDa FASP tube and digested with trypsin at 37°C overnight.

The LC-MS analysis was performed using a Orbitrap Q Exactive HF-X mass spectrometer coupled with an UltiMate 3000 LC system (Thermo Fisher Scientific, USA). A 15-cm-long LC column (i.d. 150 μm) packed with 1.9 μm C18 packing particles were used for peptide separation. The column was pulled using a micropipette puller (P-2000, Sutter Instrument, Novato, CA) for preparation of the nanoESI tips with a 5-µm opening. The spray voltage was set at 2.3 kV. Due to the high DC voltage applied, the operator should stay away from high voltage power to avoid danger. An 80-min gradient of 6%–40% buffer B (80% acetonitrile with 0.1% formic acid) was used for peptide elution. The samples were spiked with iRT standards (Biognosys) for retention time calibration. Mass spectrometry measurements were performed in data dependent acquisition (DDA) mode. The full MS scans were acquired from m/z 350-1550 at a resolution of 120 k (at m/z 200) with a target of 3e6 charges for the automated gain control (AGC) and 20 ms maximum injection time. For HCD MS/MS scans, the normalized collision energy was set to 27%, the resolution was 15 k at m/z 200, accumulated for a maximum of 30 ms or until the AGC target of 2e4 ions was reached.

For protein identification the data were searched against the *T. versicolor* proteome (annotated from the genome assembled in this study) using the Mascot search engine (Matrix science) using Genedata Expressionist software with 1% False Discovery Rate cutoff. Carbamidomethyl (C) and oxidation (M) were considered as fixed and variable modifications, respectively. MS/MS tolerance was 0.05 Da and peptide tolerance was 20 ppm. Relative protein concentrations were calculated by label free quantification from peptide volumes using a Hi3 standard method in Genedata Expressionist.

Protein intensity values were normalized across the entire dataset (21 samples) by median. Differential protein expression analysis was conducted using the R package protti (Quast et al.,2022). A fold-change of 2-fold and an adjusted *p*-value of 0.1 were adopted as the threshold for calling significant differential expression events.

### 2.6 Recombinant laccase expression and purification

The *A. oryzae* strain Cols1300 (US2019225988 A1) was used as the host for heterologous expression. Genes encoding *T. versicolor* laccases were codon optimized for *Aspergillus oryzae* (Gustafsson et al.,2004), synthesized by Azenta Life Sciences (Suzhou, China), and transformed into Cols1300 protoplasts (US 2019/0225988 A1). Recombinant laccase expression was verified by small-scale fermentation and SDS-PAGE of supernatant samples. Genomic insertion of laccase genes was verified by spore PCR and Sanger sequencing.

For recombinant laccase expression and purification, single colonies were used to inoculate slants. When it was fully sporulated, spores were collected in 5 mL of dap4C medium (WO 2021/055,395) and transferred to 3 or 4 2-L shake flasks each containing 400 mL of Dap4C-1 supplemented with 0.4 mM CuSO_4_, shaking at 30°C, 80 rpm, 4 days. The culture broths were then harvested by using a 1000 mL Rapid-Flow Bottle Top Filter 0.2-µm aPES membrane (ThermoFisher Scientific, Cat# 597–4520).

The conductivity of culture broth was adjusted to 200 mS/cm by (NH_4_)_2_SO_4_ and loaded into a Phenyl Sepharose HiTrap Butyl HP column (Cytiva) equilibrated with 20 mM Tris-HCI and 2 M (NH_4_)_2_SO_4_ at pH 7.0, then the unbounded protein was washed by the same buffer. The target protein was eluted with a gradient decrease of (NH_4_)_2_SO_4_ concentration from 2 M to 0 and the fractions with laccase activity were pooled together. The fractions with laccase activity were desalted by Sephadex G25 (Cytiva), and then loaded into a HiResQ column (Cytiva) equilibrated with 20 mM Tris-HCI at pH 8.0. The column was eluted with a gradient increase of NaCI concentration from 0 to 1 M, and the eluted fractions with laccase activity were collected, diafiltrated with 20 mM Tris-HCI at pH 7.0, then concentrated for further evaluation.

### 2.7 Syringaldazine enzymatic assay

Syringaldazine (S7896, Sigma) dissolved in 25 mM Tris-HCI at pH 7.5 with 0.05% Triton X-100 (18 μL, 0.22 mM) was mixed with 15 µL enzyme solution in 96-well plate, then shake at 30°C for 20 s. The plate was put in a microplate reader, and the absorbance at 540 nm was read with an interval of 30 s for 5 min. Calculate the value of Vmax (milli-units/min) from 5 points. The activity is determined relative to the standard curve of laccase from *Aspergillus* sp. (SAE0050, Sigma), which had been determined units at pH 7.5. One laccase unit (LAMU) is the amount of enzyme needed to convert 1 µmol Syringaldazine per minute under the given analytical conditions.

### 2.8 Benzo[a]pyrene enzymatic assay

Reduction of BaP concentration was monitored as the indicator of enzyme performance. The reaction was conducted with the following conditions: 10 mL glass tube with total solution volume at 2 mL, 20 ug laccase protein, benzo[a]pyrene at 50 μM, 1-Hydroxybenzotriazole (HBT) at 1 mM, 1% Tween 80, 10% acetonitrile, pH 5/7/9 with 50 mM Tris-HCl buffers. Meanwhile, controls with the same setup as mentioned above but without enzymes were included. After incubation at 37°C in closed tubes and dark for 24 h, for each reaction tube, 0.5 mL reaction solution was collected and mixed with 0.5 mL acetonitrile and then filtered with 0.45 µm filter paper before analysis with UPLC.

UPLC was performed with Waters ACQUITY UPLC BEH C18 column (2.1 × 100 mm, particle size 1.7 µm) to separate the reaction solutions at 50°C, with a constant flow rate of 0.4 mL/min and a mobile phase of 0.1% formate (A)/0.1% formate + acetonitrile(B) (10 min of linear gradient elution: 0 min, 60% A+40% B; 1.5 min, 60% A+40% B; 8.5 min, 100% B; 10 min, 60% A+40% B). Benzo[a]pyrene quantification was performed at 254 nm. The retention time of benzo[a]pyrene was determined to be around 7.0 min. The concentration of benzo[a]pyrene was calculated with the standard curve and the relative removal rate was estimated by the following formula: ([BaP_test]-[BaP_control])/[BaP_control]. All experiments were performed in triplicates.

## 3 Results

### 3.1 Genome assembly of the *Trametes versicolor* isolate

**FIGURE 1:**
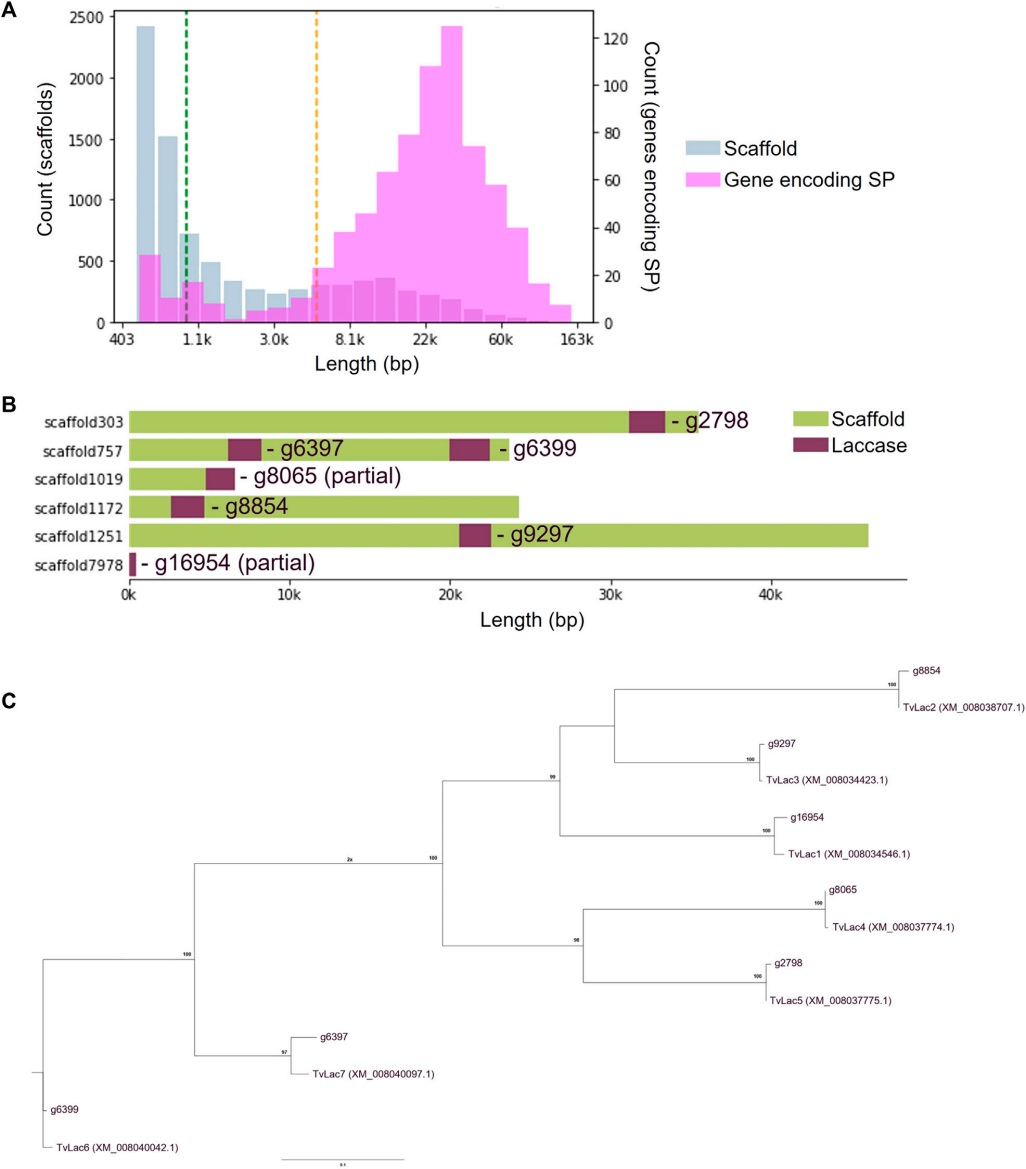
Genome assembly of the *Trametes versicolor* isolate. **(A)** Length distribution of 8,423 assembled scaffolds (Blue bar) with the mean of 5,298 bp (green dashed line) and median of 940 bp (orange dashed line), as well as distribution of 762 annotated genes encoding a secretion peptide (pink bar); **(B)** Distribution of seven laccase genes on the assembled scaffolds, including two partial sequences that were later restored by comparative genomics analysis; **(C)** Identification of laccase homologues with respective to the *Trametes versicolor* reference genome (NCBI GCF_000271585.1).

Genomic DNA was extracted from the isolate of interest, and the ITS sequence was confirmed to share 99.46% homology with the ITS sequence of *T. versicolor* (GenBank: NR_154494). Therefore, the isolate was identified as *T. versicolor*. Genome of the isolate was then sequenced and 8,423 scaffolds were assembled (Figure 1A). The genome is estimated to be 45 Mb in size containing 17,234 genes, 762 of which encode a secretion peptide. Seven laccase genes (*TvLac1-7*) were annotated (Figures 1B, C), including two partial sequences that were recovered by comparative genomics analysis.

### 3.2 Distinct laccase expression profiles induced by benzo[a]pyrene (BaP)

The *T. versicolor* isolate was grown for 5 days in liquid media before the addition of 10 mg/L benzo[a]pyrene (BaP) as both the inducer and substrate, or sterile water as mock treatment. The cultures were sampled 0, 1, 4 and 11 days after the BaP treatment (Supplementary Figure S1), corresponding to 5, 6, 9 and 16 days upon inoculation. The first two sampling time points corresponded to the lag growth phase, while the third sampling time point corresponded to the logarithmic growth phase and the last sampling time point corresponded to the stationary growth phase. Transcriptomes and secretomes (secreted proteomes) were analyzed by RNA-seq and mass spectrometry respectively. Transcripts of all seven laccases (*TvLac1-7*) were detected in the transcriptomes. The TvLac2, TvLac3, TvLac5 and TvLac6 laccase isoforms were detected in the secretomes, whereas the TvLac1, TvLac4 and TvLac7 isoforms were non-detectable. Laccase expression was quantified at both transcript and protein levels displaying distinct profiles (Supplementary Tables S1, S2).

**FIGURE 2:**
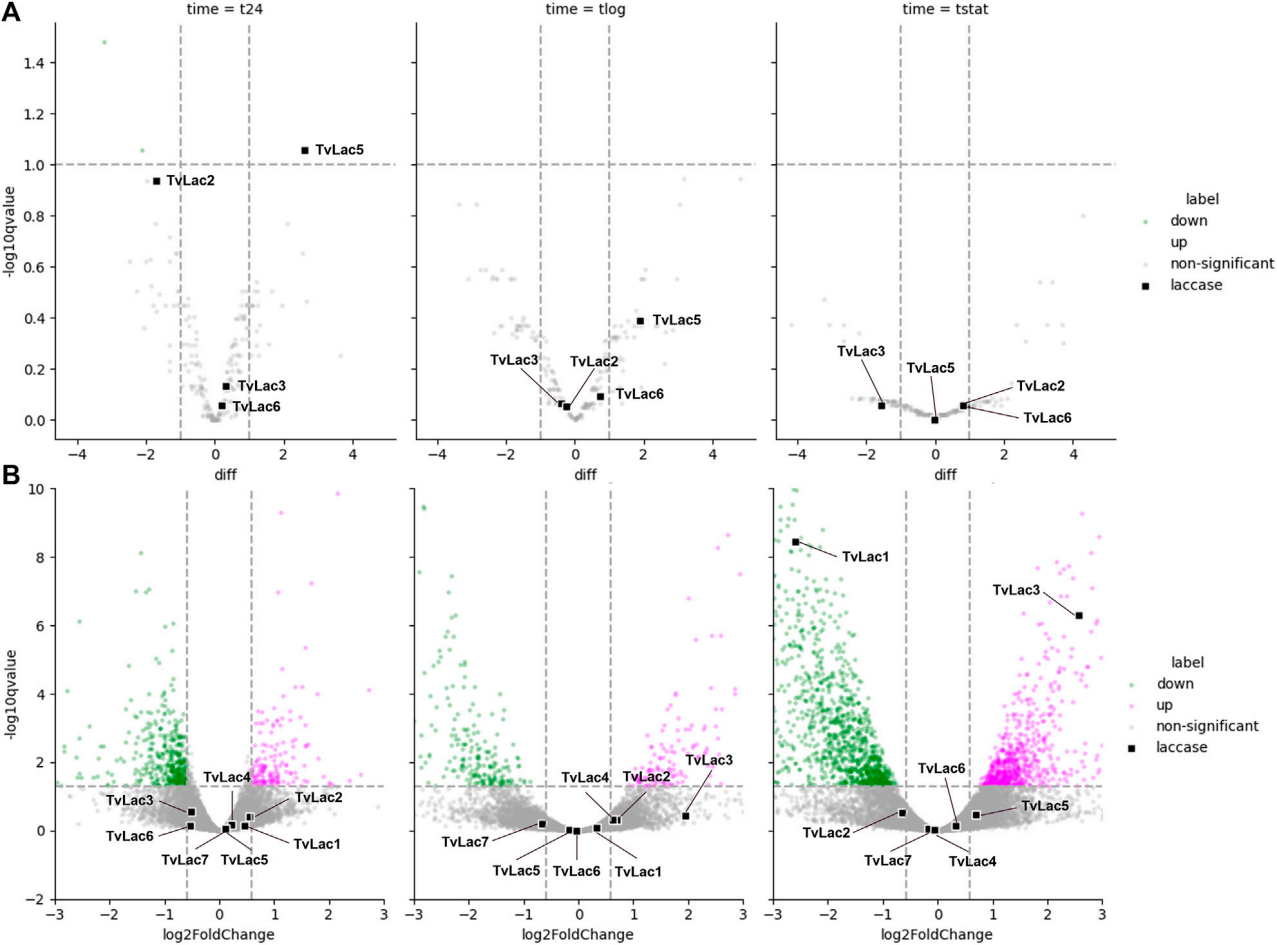
Differential laccase expression. **(A)** Differential expression of laccases in the secretome quantified by mass spectrometry 1 (t24), 4 (tlog), 11 (tstat) days after BaP treatment, with laccases highlighted as black squares; The other proteins significantly downregulated (adjusted *p*-value <0.05) are labelled in green, while no other proteins are significantly upregulated; **(B)** Differential expression of laccases in the transcriptome quantified by RNA-seq 1 (t24), 4 (tlog), 11 (tstat) days after BaP treatment, with laccases highlighted as black squares, and other transcripts significantly upregulated or downregulated (adjusted *p*-value <0.05) labelled in magenta and green, respectively; The x- and *y*-axis are zoomed to clearly show all the laccases.

In general, the transcriptomic response of laccase genes is delayed in the time course. Specifically, expression of all seven laccase genes remained unchanged one and 4 days after BaP treatment. Expression of *TvLac3* gene was upregulated (5.91-fold, adjusted *p*-value <0.05) 11 days after the BaP treatment, and expression of *TvLac1* gene was downregulated (0.16-fold, adjusted *p*-value <0.05) (Figure 2B), while all the other five laccase genes remained unchanged.

**FIGURE 3:**
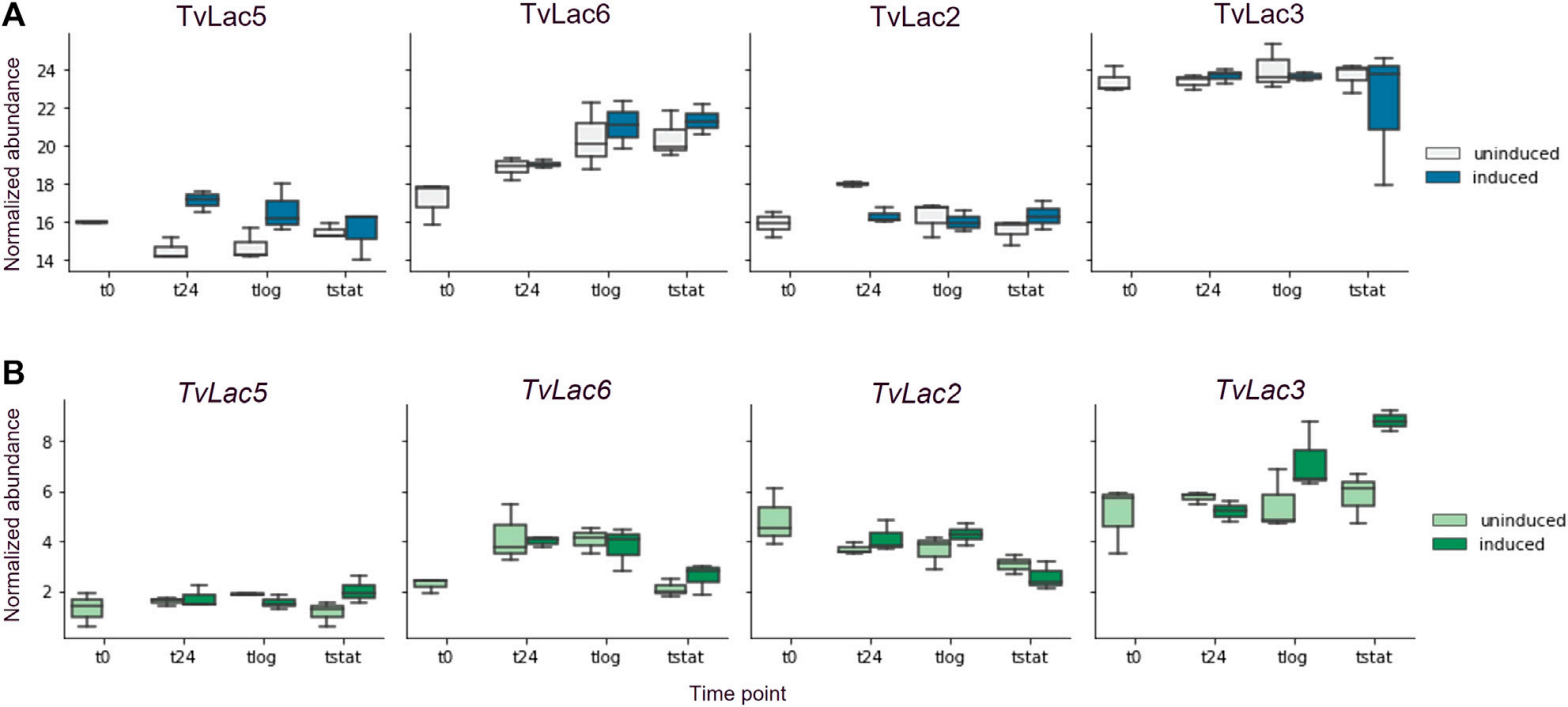
Time-course laccase expression. **(A)** Time-course protein abundance quantified by mass spectrometry, uninduced (light blue) and induced (dark blue) by BaP; **(B)** Time-course transcript abundance quantified by RNA-seq, uninduced (light green) and induced (dark green) by BaP; Each box shows the quartiles of normalized abundance with n = 3.

Contrary to the delayed transcriptomic response, the proteomic response to BaP was rapid. Specifically, protein abundance of the TvLac5 isoform was upregulated (2.6-fold, adjusted *p*-value <0.1) in the secretomes 1 day after BaP treatment (Figure 2A), and gradually decreased to the pre-induction level at the stationary phase (Figure 3A). Oppositely, protein abundance of the TvLac2 isoform showed a tendency of being downregulated 1 day after BaP treatment (adjusted *p*-value 0.1154) and was maintained at low level since. Without BaP treatment, protein abundance of the TvLac5 isoform decreased to the lowest level before the logarithmic growth phase, whereas protein abundance of the TvLac2 isoform peaked before the logarithmic growth phase and then decreased. Taken together, BaP treatment rapidly altered the expression patterns of both TvLac5 and TvLac2 isoforms in the secretomes, whereas protein abundance of the TvLac3 and TvLac6 isoforms was not responsive to BaP.

Peroxidases constituting the lignin degrading enzyme system of *T. versicolor*, including lignin peroxidases, versatile peroxidases and manganese peroxidases, did not show induction in response to BaP treatment (Supplementary Figure S2).

Taken together, it is hypothesized that the TvLac5 isoform contributes to the BaP-degrading activity from the *T. versicolor* isolate, thus possessing significant enzymatic activity against BaP.

### 3.3 Lack of correlation between transcriptomes and secretomes

No significant correlation was detected between the whole transcriptomes and secretomes at any sampling time points (Supplementary Figure S3). The correlation was then examined for individual laccases. Despite being responsive to BaP treatment at protein level, no corresponding expression response was detected at transcript level for both *TvLac5* and *TvLac2* (Figure 3B), indicating that the rapid BaP responses were likely not regulated by transcription. Protein abundance of the TvLac3 isoform was maintained at high level across the entire growth phase independent to BaP treatment (Figure 3A). At the transcript level, *TvLac3* was induced 11 days after BaP treatment possibly to maintain its high-level protein expression, indicating that the delayed BaP response was likely regulated by transcription. Protein abundance of the TvLac6 isoform first increased until the logarithmic phase, and then was maintained at the stationary phase, also independent to BaP treatment (Figure 3A). Correspondingly, the transcript abundance of TvLac6 gradually increased until the logarithmic phase and decreased at stationary phase (Figure 3B), indicating that the BaP-independent expression was likely regulated by transcription.

### 3.4 A laccase from *Trametes versicolor* displays high BaP-degrading activity

**FIGURE 4:**
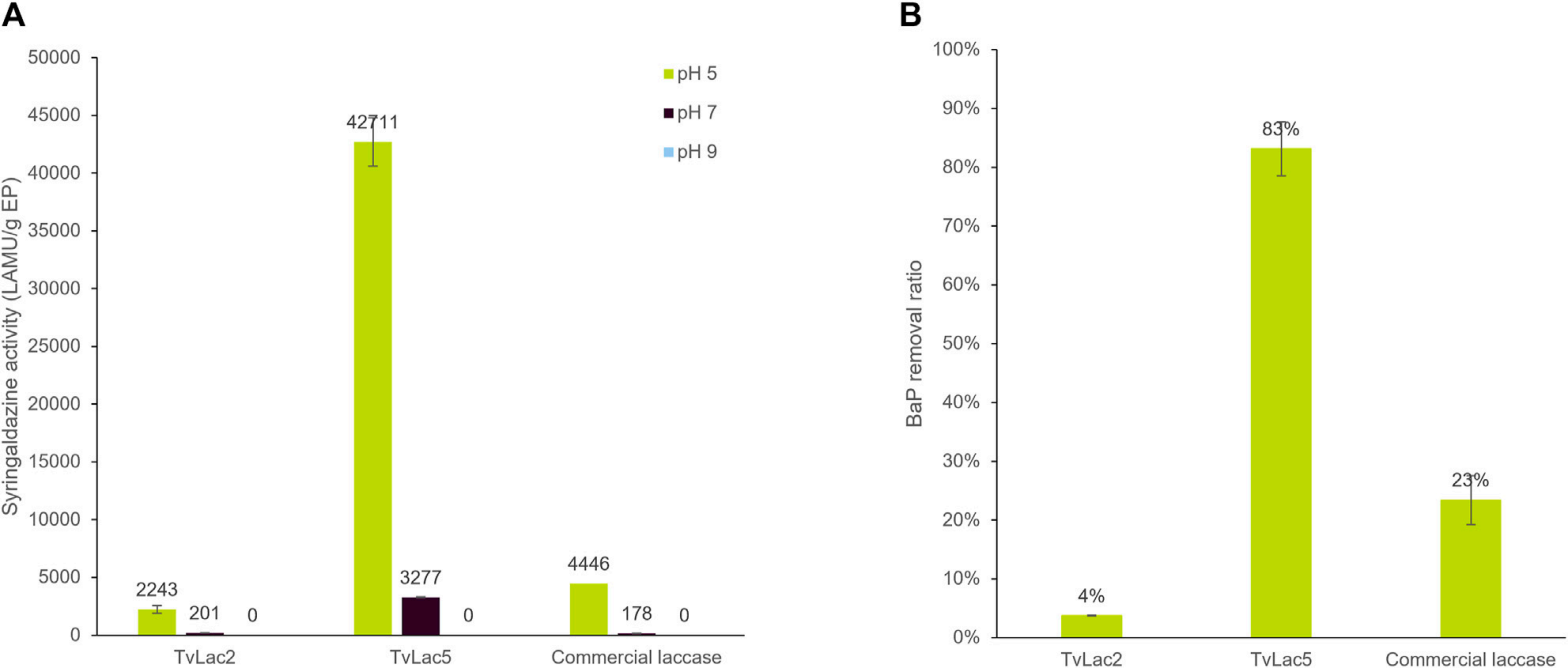
Biochemical characterization of identified laccases. **(A)** Activity of laccase TvLac2, TvLac5 and the commercial laccase product (Sigma 38429) against syringaldazine at pH 5, 7, and 9; Error bars indicate standard deviation with n = 2. **(B)** BaP-degrading activity of laccase TvLac2, TvLac5 and the commercial laccase product at pH 5; Error bars indicate standard deviation with n = 3.

Recombinant TvLac5 and TvLac2 laccases were successfully expressed in *A. oryzae* and purified (Supplementary Figure S4). The purified enzymes were assayed against syringaldazine, a generic substrate of laccases, as well as against benzo[a]pyrene (BaP). All three laccase enzymes showed the highest activity against syringaldazine at pH 5, with moderate activity at pH 7 and non-detectable activity at pH 9, indicating that these laccases perform the best under acidic conditions. Therefore, BaP-degrading activities were only measured at pH 5 in the following experiments. In general, recombinant TvLac5 showed higher activities compared to TvLac2 and the commercial *T. versicolor* laccase product (Sigma 38,429). Specifically, TvLac5 showed 19-fold and 9.6-fold higher activity against syringaldazine (pH 5) compared to TvLac2 and the commercial laccase respectively (Figure 4A). More importantly, TvLac5 also showed 21-fold and 3.6-fold higher activity against BaP compared to TvLac2 and the commercial laccase respectively (Figure 4B). Taken together, TvLac5 and TvLac2 show distinct activity profiles against syringaldazine and BaP, and that TvLac5 outperforms the commercial laccase product on BaP degradation.

## 4 Discussion

### 4.1 Multi-omics profiling-guided approach–values and limitations

Multi-omics profiling is a valuable approach for identification of high-performing enzymes. As demonstrated in this study, the proteomics analysis led to the key laccase candidate, whereas the transcriptomics analysis provided essential clues to laccase expression regulation.

However, there are also limitations to this approach. First, identification of an effective inducer is critical, since often the substrate itself may not be sufficient. For example, it was previously reported that co-substrate was required to induce significant activity from *Trametes gibbosa* (Wen et al., 2011). Without effectively induced activities, it is unlikely that responding enzymes would be detected with robust sequencing signals. In this study, co-substrates were not required for BaP degradation, yet may offer boosting effects in future studies. Second, the correlation between transcriptomics and proteomics results may be ambiguous. It was previously observed that protein abundance may not necessarily correlate with transcript abundance on the whole-omics level in yeast (Gygi et al., 1999). Instead, individual proteins and transcripts should be considered. In this study, proteomics data provided the most direct evidence addressing the scientific questions. Third, there are potentially subtle yet biologically significant signals that could not be resolved by current analytical and statistical approaches. Especially when the chemical processes contain multiple steps, only the enzymes involved in the initial step may be identified with high confidence. Bio-catalyzed PAH degradation is a highly complicated multi-step reaction (Ghosal et al., 2016). The laccase identified in this study by the multi-omics approach is likely to only facilitate the initial step of catalysis, while other enzymes involved in the following steps could not be detected. Time-course sampling can mitigate some of the above limitations and facilitate the identification of expression patterns. Heterologous expression followed by biochemical validation is also essential.

### 4.2 Implications on differential expression and regulation patterns between laccase genes

It has been elucidated that fungal ligninolytic enzyme isoforms encoded by homologous genes possess diversified native functions during development (Sakamoto et al., 2005) and defense (Pezet et al.,1991). The homologous genes are also expressed in various patterns in response to environmental signals, such as nutrients concentration, temperature, and light (Ramírez et al., 2010; Yang et al., 2017). It has also been reported that fungal laccase activity could be induced by organic contaminants, such as PAH (Wen et al., 2011). However, it is less clear how the laccase homologues differentially contribute to the response, due to lack of systematic comparisons between all laccase homologues at both transcript and protein level in time course.

In this study, coordinated expression between laccases encoded by the *T. versicolor* isolate upon BaP treatment was elucidated and was in consistency with their functional diversity against the BaP substrate. Moreover, laccase homologues appear to adopt distinct expression regulation mechanisms at not only transcriptional but also post-transcriptional level. The emerging pattern from this study is that at least four out of the seven laccases encoded in the *T. versicolor* isolate are expressed extracellularly. Under normal growth conditions, TvLac2, TvLac3 and TvLac6 could be responsible for the native functions, such as lignin degradation. Under BaP treatment which implies a stressed condition, TvLac5 could be regulated post-transcriptionally for rapid mitigation response. TvLac1, TvLac4 and TvLac7 may function intracellularly, in analogy to enzymes functioning in the intracellular lignin catabolism pathway (del Cerro et al., 2021), therefore not detected in the secretomes.

### 4.3 Potential for product development

It is extensively reported that laccase can be applied as an biocatalytic tool for degrading recalcitrant pollutants such as PAHs in water or soil, yielding less-toxic compounds (Kadri et al., 2017). Laccase has also been proved effective to boost the fast growth of externally applied heavy-oil degrading bacteria, by oxidizing the phenols and aromatic compounds firstly and hence removing the inhibition to the microbial community (Dai et al., 2020). Therefore, the combined use of laccase and microbes have the great potential to be implemented as an eco-friendly bio-remediation strategy for removing broad spectrum of petroleum derived pollutants in the environment, which is in great demand for the sustainable society development.

However, most of the reported studies stay in research or pilot scale, and large-scale commercial application cases of using laccase for water or soil remediation are still very few. Two critical challenges which have limited the industrial scaling up of the laccase application are summarized as 1) lack of commercial laccase products that are both specially developed for pollutant degradation purposes and also viable for low-cost production at industrial scale; 2) challenging environmental factors (inhibitors, poor accessibility to substrates, broad pH conditions, etc.) negatively impacting the stability and efficacy of enzymes in the real water and soil environments (Arregui et al., 2019).

In this study, we have identified TvLac5 from *T. versicolor* which shows both prominent activity and great ability to be industrially produced, which are the key factors to be considered for developing commercial enzyme products. The potential for TvLac5 as the next-generation laccase product was validated, as recombinant TvLac5 showed significantly higher activity against both syringaldazine and BaP compared to the benchmark commercial laccase having been tested. Moreover, secretory expression of recombinant TvLac5 are successfully achieved in the industrial host *A. oryzae*. The establishment of this heterologous expression system has great advantages of low-cost downstream processing than intracellular microbial production of laccases and hence shows significant potential for enzyme product development and future commercialization (Välimets et al., 2023).

Looking forward, to realize the successful application of laccase for bio-remediation, the development of scalable and viable application process to ensure stability, efficacy and reusability of enzymes are also very critical. In this direction, various technologies for enzyme immobilization pose as very useful tools to overcome the challenging factors in the environmental remediation process and to achieve effective application of the used laccase products (Wang et al., 2023). Therefore further development is envisioned with an immobilized version of TvLac5 in this study.

## Data Availability

The datasets presented in this study are deposited in the NCBI repository, accession number BioProject PRJNA1014135 (https://www.ncbi.nlm.nih.gov/bioproject/PRJNA1014135).
