# Supplementary material for: Distinct laccase expression and activity profiles of Trametes versicolor facilitate degradation of benzo[a]pyrene
Source: Front Bioeng Biotechnol. 2023 Sep 21;11:1264135. doi: 10.3389/fbioe.2023.1264135 (PMC10551628; doi:10.3389/fbioe.2023.1264135)
Supplement: Supplementary file 3 [file DataSheet1.docx]

Supplementary Material

# Supplementary Figures
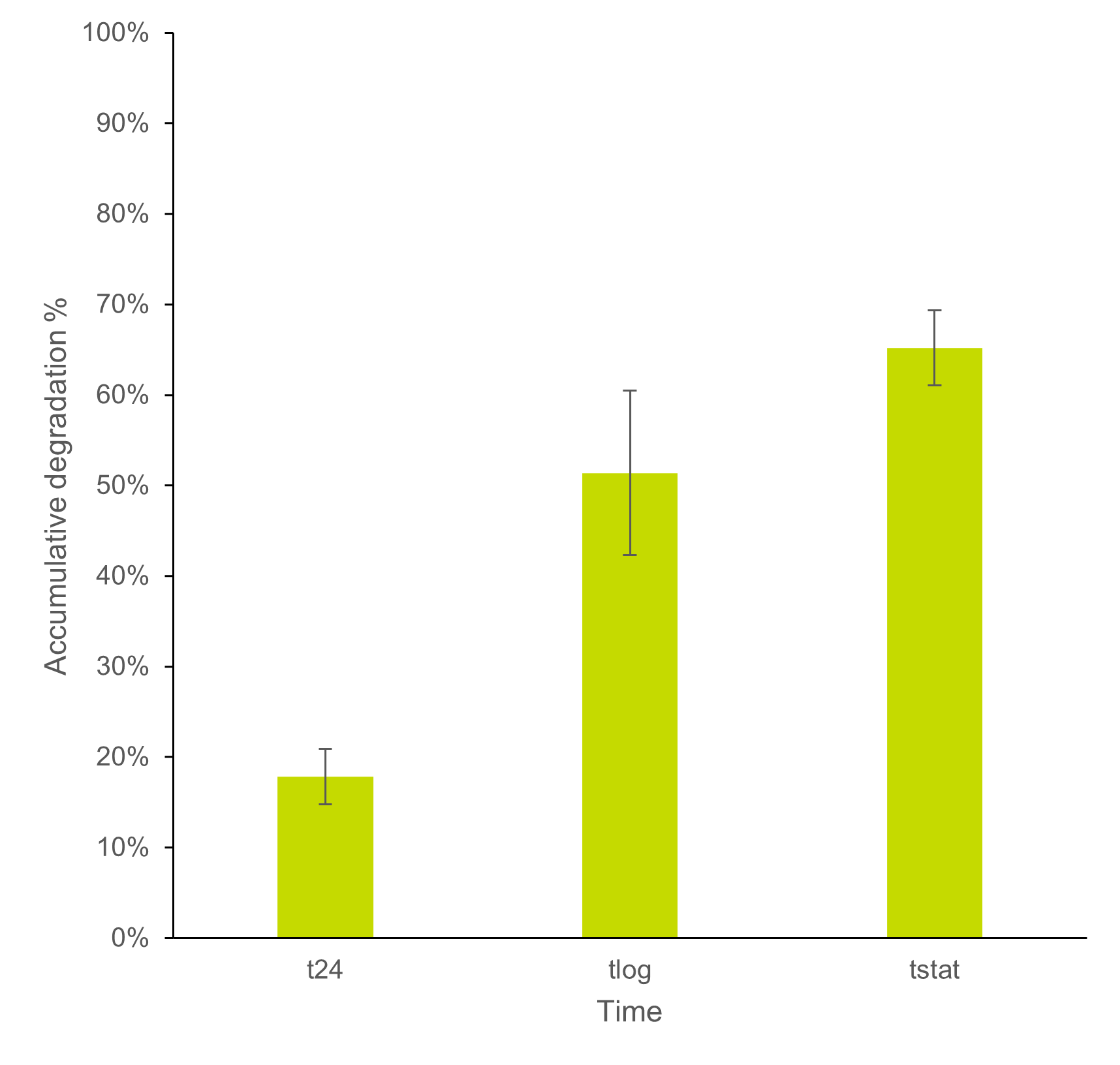


**Supplemental Figure 1.** BaP degradation by *Trametes versicolor*. Accumulative BaP degradation rate at 1 (t24), 4 (tlog), 11 (tstat) days after BaP treatment.


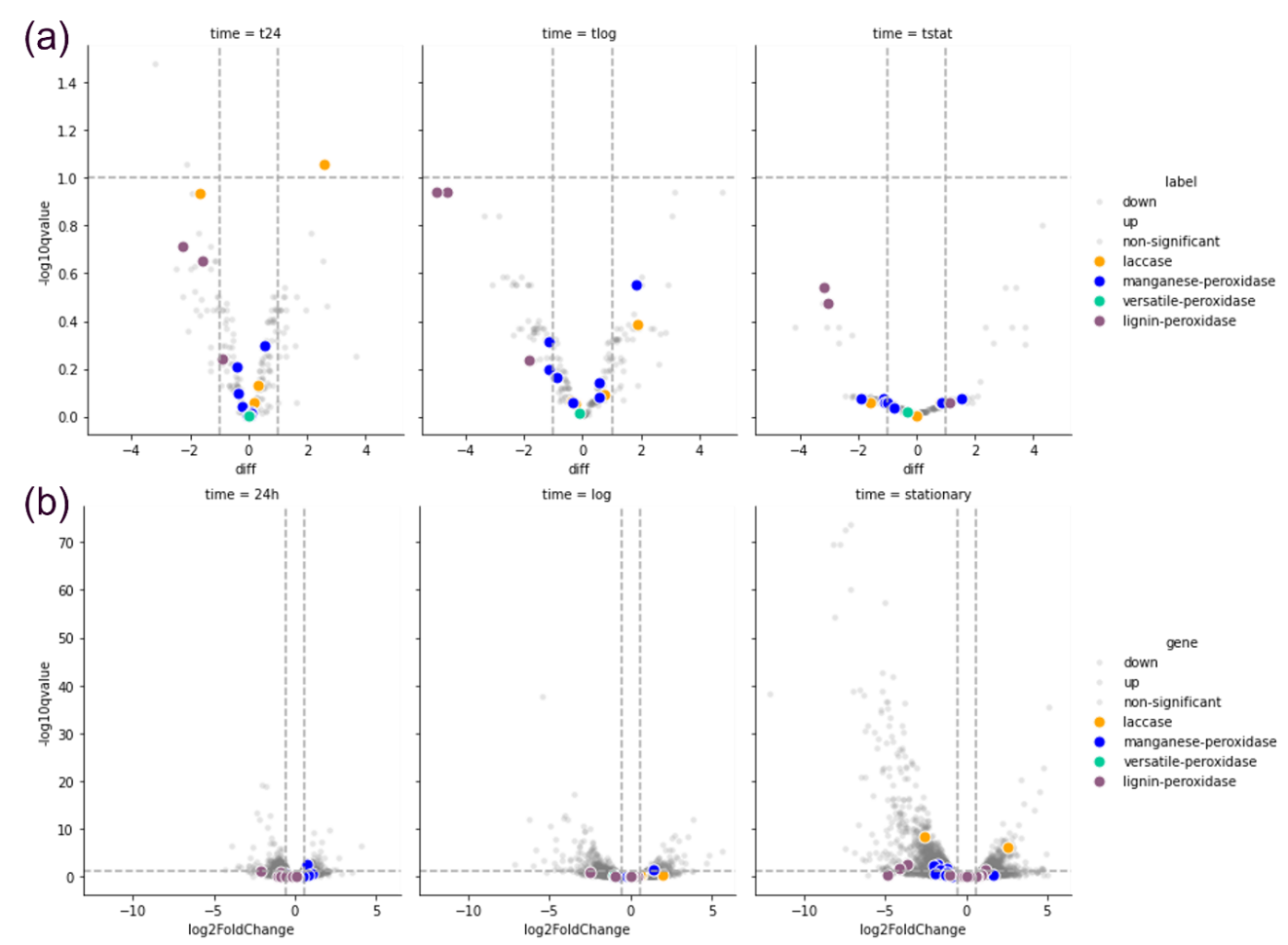


**Supplemental Figure 2.** Differential peroxidase expression. Differential expression of peroxidases and laccases 1 (t24), 4 (tlog), 11 (tstat) days after BaP treatment in the secretome sequenced by mass spectrometry (a) and transcriptome sequenced by RNA-seq (b), with laccases highlighted in orange, manganese peroxidases highlighted in blue, versatile peroxidases highlighted in green and lignin peroxidases highlighted in purple.


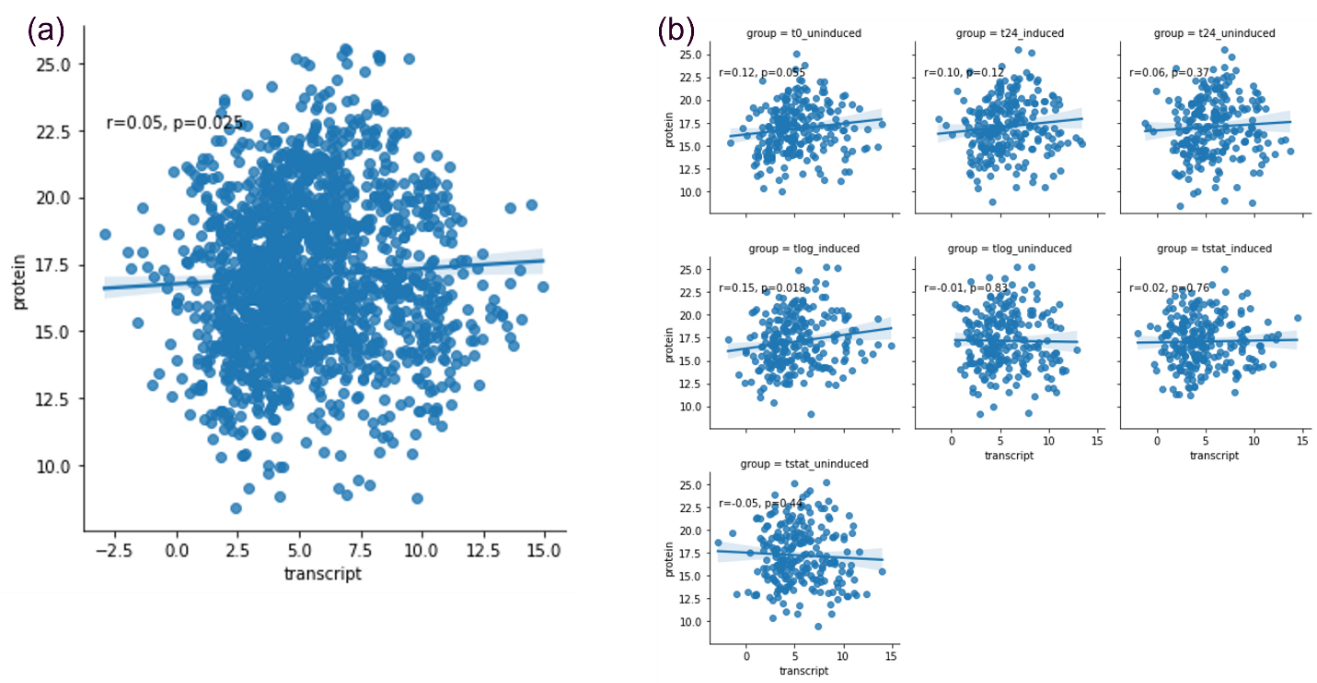


**Supplemental Figure 3.** Correlation between protein and transcript abundance. (a) Normalized protein abundance was plotted against normalized transcript abundance for each identified transcript-protein pair; (b) Correlation data in (a) split by sampling groups.


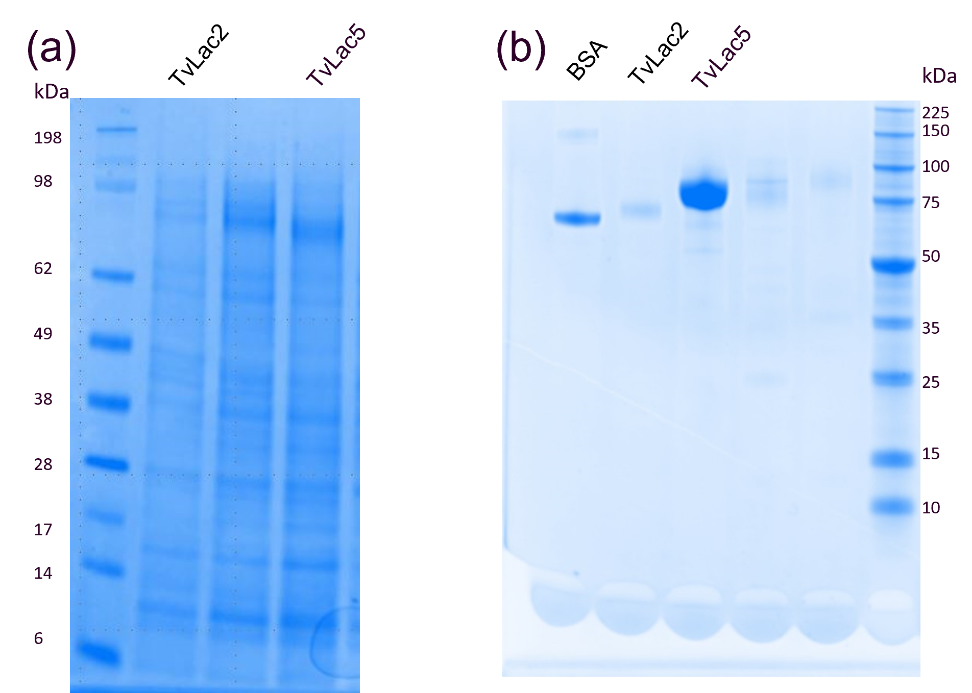


**Supplemental Figure 4.** Recombinant laccase expression and purification. (a) SDS-PAGE of supernatant samples yielded from recombinant laccase expression in *Aspergillus oryzae* (Cols1300); (b) SDS-PAGE of purified recombinant laccase samples.

# Supplementary Tables

**Supplementary Table 1.** Transcript abundance. Fold change and statistics of normalized transcript abundance

**Supplementary Table 2.** Protein abundance. Fold change and statistics of normalized protein abundance
